# Evaluating Three-Dimensional Navigation versus Conventional Fluoroscopy for Posterior Cervical Foraminotomy: An Exploratory Cohort Comparison

**DOI:** 10.1055/a-2790-3799

**Published:** 2026-07-01

**Authors:** Christian Blume, Andrej Bitter, Guido Wapenhans, Patrick Weidle, Kerstin Jütten, Miguel Pishnamaz, Alexander Romagna, Ulf Bertram, Christian Andreas Mueller, Hans Clusmann, Tobias Philip Schmidt

**Affiliations:** 1 Department of Neurosurgery, University Hospital RWTH Aachen, Aachen, North Rhine-Westphalia, Germany; 2 Department of Orthopedics and Trauma Surgery, Neuwerk Hospital Mönchengladbach, Mönchengladbach, North Rhine-Westphalia, Germany; 3 Department of Orthopaedics, Trauma and Reconstructive Surgery, University Hospital RWTH Aachen, Aachen, North Rhine-Westphalia, Germany; 4 Department of Neurosurgery, München Klinik Bogenhausen, Munich, Bayern, Germany

**Keywords:** posterior cervical foraminotomy, 3D navigation, fluoroscopy, intraoperative CT, foraminal stenosis

## Abstract

**Background:**

Unilateral monosegmental radiculopathy, caused by foraminal soft disc nerve root compression, can be treated with posterior cervical foraminotomy (PCF). Conventional fluoroscopy is widely used for level localization and verifying decompression extent intraoperatively. However, the failure rate increases, especially in obese patients and the lower cervical spine. Can intraoperative three-dimensional (3D) navigation improve outcomes in PCF compared with conventional fluoroscopy?

**Materials and Methods:**

In this retrospective study, we analyzed two groups: 42 patients (mean age: 54 ± 10 years) who underwent PCF using intraoperative 3D navigation (study group) and 63 patients (mean age: 51 ± 11 years) who underwent PCF with conventional fluoroscopy (control group). Each cohort was divided into upper (C3–C6) and lower cervical spine (C6–T1) subgroups. Differences were explored by Mann–Whitney U and Friedman tests.

**Results:**

Both groups experienced significant postoperative symptom improvement, with no significant neurological differences. Subgroup analyses revealed no significant differences between surgeries in the upper (C3–C6) and lower (C6–T1) cervical spine. Only blood loss in the C3–C6 group differed significantly between control and study groups.

**Conclusion:**

Intraoperative 3D navigation was successfully used for PCF, resulting in significant symptom relief. Outcomes after PCF with 3D navigation appeared comparable to those achieved with conventional fluoroscopy, without statistical evidence of a clear advantage. Thus, technical complexity should be considered when selecting the surgical method.

## Introduction

Posterior cervical foraminotomy (PCF) is a possible procedure to treat soft cervical disc herniations in the neuroforamen causing radicular syndromes. 1 2 3 It has advantages over the ventral approach, as it can help to avoid complications such as postoperative dysphagia and recurrent laryngeal palsy. In addition, PCF can help preserve the range of motion in the treated segment and reduce stress on the adjacent levels, provided that the extent of decompression remains as minimal as needed. 4 5 6 7 To enter and decompress the foraminal space, the surgeon applies a bony window to the hemilamina. 2 Traditional fluoroscopy has always been used to identify the level of the desired neuroforamen. A lateral X-ray in most cases can easily visualize the vertebrae C1–C4. Shoulder retraction can help locate the segment C4/C5 and lower segments, but there is a strong dependence on physiognomy from C6 downward: The quality of summation images decreases as the neck and shoulder contribute more tissue to the X-ray beam, making it progressively harder to visualize the lower cervical spine and thus accessing the surgical site. 8 9 Three-dimensional (3D) navigation is a technology that uses X-rays to collect a dataset that can be converted into 3D images. 10 This visualization is comparable to a computed tomography (CT) dataset. Special software allows virtual visualization of surgical instruments on images, aiding orientation. Therefore, 3D navigation has been found to have advantages over fluoroscopy in terms of surgery time, reduced length of stay, radiation exposure, and complication rates. 11 12 13 14 To our knowledge, no studies have investigated the impact of the advantage of 3D navigation on surgery time, length of hospital stay, and neurological outcomes in the context of upper versus lower cervical spine surgery. Therefore, we conducted a retrospective cohort study to explore the use of 3D navigation in PCF and specifically examine the differences between the upper and lower cervical spine.

## Materials and Methods

### Study Procedure and Description of the Study Group

The Local Ethics Committee of the Medical Faculty of the RWTH Aachen University (EK 23-062) approved this retrospective cohort study. Data were analyzed anonymously. Between August 2017 and April 2022, 44 patients underwent PCF assisted by 3D navigation using Medtronic O-arm System technology. The main presenting symptoms of this patient cohort were unilateral radicular arm pain, with some patients experiencing motor weakness. Neck and radicular pain were evaluated using a numeric rating scale (NRS 0–10, with 0 = no pain and 10 = severest pain), and paresis was assessed using the Janda muscle strength scale (0–5, with 0 = complete paralysis and 5 = full muscle strength). Preoperative imaging revealed lateral disc herniation and/or foraminal stenosis in one or two segments. 3D navigation was employed in all procedures of the study group to assist the surgeon in orientation and achieving full root decompression. Due to the increasing difficulty in visualizing the lower cervical spine and accessing the surgical site, we divided the sample into two groups of patients with disc herniations in the upper and lower cervical spine (C3–C6: *n* = 25; C6–T1: *n* = 17, *p* = 0.530). Two patients were excluded from statistical analysis because the necessary data were incomplete (remaining patient number, *n* = 42).

A historical patient cohort ( *n* = 63) serves as a control group since these patients did not receive navigated surgery, but instead underwent foraminotomy using conventional X-ray fluoroscopy. This cohort has already been studied to compare its outcomes with those of an anterior cervical discectomy and fusion (ACDF) cohort. 15

### Surgical Procedure

The patient is placed in a prone position under general anesthesia, with the head secured using a Mayfield clamp. Before draping, a 3D radiographic dataset is acquired to identify the decompression level. The StealthStation Treon System (Medtronic, Medtronic, Minneapolis, Minnesota, US) is used for navigation, employing an infrared camera and passive markers on surgical instruments. The entry point is determined using sagittal images to target the facet joint and axial images to aim at the laminofacet junction. A 1.5-cm skin incision is made, followed by sharp opening of the nuchal fascia and subperiostal release of the paravertebral muscles. The bony anatomy is exposed using the Medicon “Piccolino” retractor system. Navigation accuracy is verified by probing bony landmarks. A high-speed drill with a diamond burr is used for the foraminotomy, with the navigation probe confirming placement. After removing the ascending facet and portions of hemilaminae, soft tissues covering the neuroforamen are bluntly dissected. The ligamentum flavum is removed with hooks and punches to expose the nerve root. Following decompression, a final inspection is done with a blunt hook. The wound is then closed in layers.

In the control group, the surgical steps of a foraminotomy were performed similarly; however, the incision size used for a mini-open approach was approximately 4.0 cm and the intraoperative imaging control was different. X-ray fluoroscopy with a C-arm was used, and imaging was conducted in both lateral and anterior–posterior planes.

All procedures in the navigation group were performed by a single senior spine surgeon, whereas the control group included multiple senior spine surgeons from the same institution. This may introduce a performance bias.

### Outcomes

Due to the retrospective nature of the study and limited availability of standardized patient-reported outcome measures (e.g., Neck Disability Index, EQ-5D), neurological improvement was operationalized using motor function (Janda scale) and pain intensity (NRS). The minimal clinically important difference (MCID) for this outcomes were defined as an increase of ≥1 points on the Janda scale and ≥2 points on the NRS scale. 16 Secondary outcomes included surgery time and hospital stay duration. The MCID for these outcomes was defined as a reduction of ≥20 minutes for surgery time and ≥2 days for hospital stay.

### Data Analysis

All statistical analyses were performed using IBM SPSS Statistics (Version 27). Group differences in age and body mass index (BMI) were analyzed by independent samples *t* -tests. Sex and major comorbidities (diabetes, high blood pressure, nicotine addiction, depression) were analyzed by Pearson's chi-square tests. Between-group differences in neurological outcome (neck pain, radicular pain, and paresis) for each time point (pre-, postoperative, follow-up) were explored by Mann–Whitney U tests. Within-group longitudinal differences in neurological outcome from pre- to postoperative and follow-up examination were analyzed for each subgroup by Friedman tests of differences among repeated measures. Analogous to the analyses of the cohort operated on with intraoperative CT navigation, we analyzed demographics and between-group differences in neurological outcome in the historical control cohort using conventional X-rays. Longitudinal pre-/postoperative differences in neurological outcomes were examined using the Wilcoxon signed-rank test. In the control group, data are available for the pre- and postoperative time points, but complete follow-up data are not available. Effect sizes, including 95% confidence intervals, were calculated for all group comparisons.

## Results

### Demographics and Preoperative Clinical Status of the Study Group

Baseline characteristics (age and gender) and relevant comorbidities (diabetes, high blood pressure, nicotine addiction, depression) are shown in Table 1 for the whole sample ( *N* = 42) and for subgroups. Subgroups did not differ with regard to age ( *p* = 0.293), BMI ( *p* = 0.144), diabetes ( *p* = 0.672), high blood pressure ( *p* = 0.209), nicotine addiction ( *p* = 0.752), or depression ( *p* = 0.632).

**Table 1:** Baseline characteristics showing demographics, major comorbidities, and clinical findings for the whole sample of included patients in the study group and for subgroups

	Overall ( *n* = 42)		C3–C6 ( *n* = 25)		C6–T1 ( *n* = 17)		C3–C6 vs. C6–T1
	*M*	SD	*M*	SD	*M*	SD	*p*
Age (y)	54.2	9.6	55.5	9.9	52.2	9.0	0.293
BMI	29.0	6.7	27.7	5.2	30.9	8.1	0.144
Neck pain preop	7.5	2.4	7.6	2.2	7.4	2.6	0.947
Radicular pain preop	7.7	2.0	7.8	1.8	7.6	2.3	0.864
Paresis preop	4.1	1.1	4.4	0.9	3.7	1.2	**0.033**
Gender (f, m)	21, 21		14, 11		7, 10		0.530
Diabetes (y, n)	6, 36		3, 22		3, 14		0.672
High blood pressure (y, n)	19, 23		9, 16		10, 7		0.209
Nicotine addiction (y, n)	22, 19		12, 12		10, 7		0.752
Depression (y, n)	5, 37		2, 23		3, 14		0.632

Abbreviations: BMI, body mass index; f, female; m, male; M, mean; preop, preoperative; SD, standard deviation; y, yes.

Note: Quantification of pain according to numeric rating scale (NRS). Quantification of muscle strength according to Janda.

With regard to the neurological status, no significant preoperative differences were found in neck or radicular pain ( *p* = 0.947 and *p* = 0.864) between subgroups, but the severity of paresis was higher in patients with disc herniation in the lower as compared with upper cervical spine ( *M _C3–C6_* = 4.4 and *M _C6–T1_* = 3.7, *U* = 135.50, *p* = 0.033).

### Demographics and Preoperative Clinical Status of the Control Group

Baseline characteristics (age and gender) and relevant comorbidities (diabetes, high blood pressure, nicotine addiction, depression) are presented in Table 2 (sample size, *N* = 63) and for subgroups. The subgroups showed no significant differences in age ( *p* = 0.255), BMI ( *p* = 0.137), diabetes ( *p* = 0.100), high blood pressure ( *p* = 0.053), nicotine addiction ( *p* = 0.615), or depression ( *p* = 0.600). Similarly, no significant preoperative differences were found in terms of preoperative pain ( *p* = 0.083) or preoperative paresis ( *p* = 0.134).

**Table 2:** Baseline characteristics showing demographics, major comorbidities, and clinical findings for the whole sample of included patients in the control group and for subgroups

	Overall ( *n* = 63)		C3–C6 ( *n* = 17)		C6–T1 ( *n* = 46)		C3–C6 vs. C6–T1
	*M*	*SD*	*M*	*SD*	*M*	*SD*	*p*
Age (y)	50.9	10.5	48.4	10.2	51.8	10.6	0.255
BMI	25.5	4.3	27.4	6.1	24.9	3.4	0.137
Pain preop	8.7	1.4	9.2	0.9	8.5	1.5	0.089
Paresis preop	4.3	0.7	4.5	0.5	4.2	0.7	0.172
Gender (f, m)	27, 36		9, 8		18, 28		0.395
Diabetes (y, n)	6, 57		2, 15		4, 42		0.519
High blood pressure (y, n)	14, 49		1, 16		13, 33		0.088
Nicotine addiction (y, n)	7, 56		2, 15		5, 41		0.615
Depression (y, n)	2, 61		0, 17		2, 44		0.600

Abbreviations: BMI, body mass index; f, female; m, male; M, mean; preop, preoperative; SD, standard deviation; y, yes.

Notes: Quantification of pain according to numeric rating scale (NRS). Quantification of muscle strength according to Janda.

### Between-Group Differences of the Study Group in Neurological Outcome at Postoperative Discharge and Follow-up

**Fig. 1:**
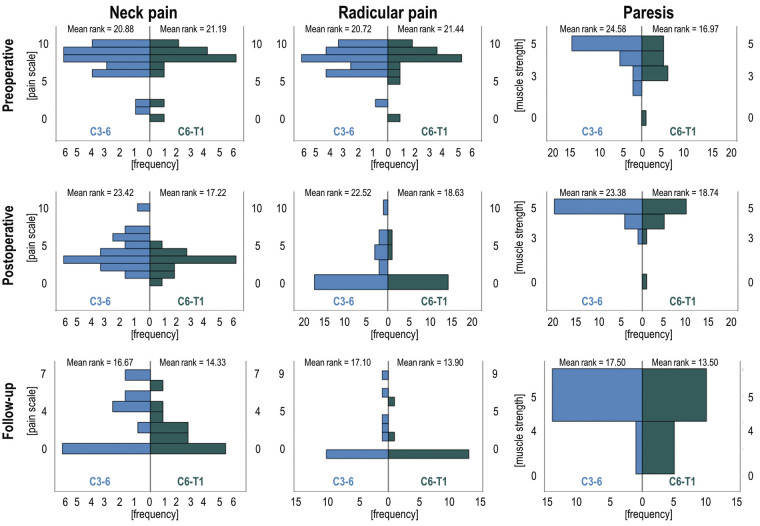
Neurological outcomes of the study group: preoperative, postoperative, and follow-up comparison between and within both subgroups (C3–C6 in blue, C6–T1 in green). Mean ranks were calculated through subgroup comparison. Please note that no comparison is significantly different (all *p* > 0.05): postop = postoperative at discharge, F/U = follow-up. Quantification of pain according to numeric rating scale (NRS). Quantification of muscle strength according to Janda.

Subgroups did not differ significantly with regard to the time of surgery or their length of stay ( *p* = 0.987 and 0.869, respectively). Similarly, no significant subgroup differences were found with regard to postoperative and follow-up neurological status (all *p* > 0.05, Fig. 1 ).

### Between-Group Differences of the Control Group in Neurological Outcome at Postoperative Discharge and Follow-up

**Fig. 2:**
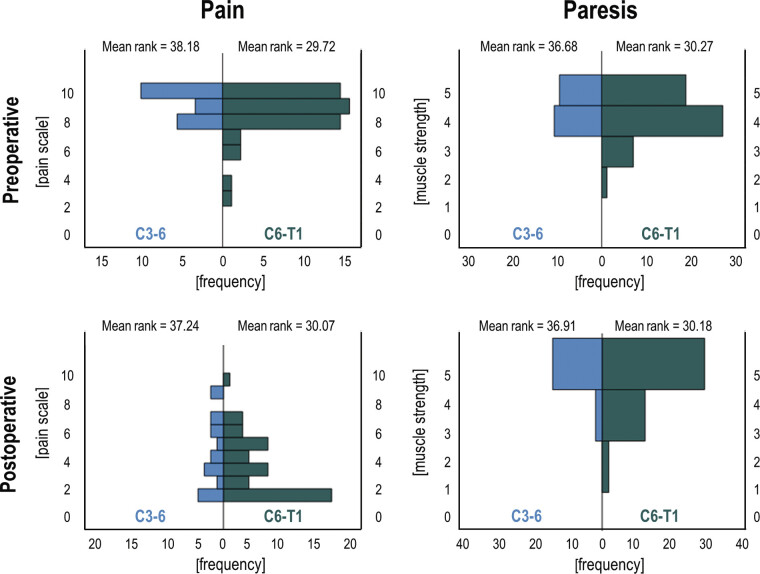
Neurological outcomes of the control group: preoperative and postoperative comparison between and within both subgroups (C3–C6 in blue, C6–T1 in green). Mean ranks were calculated through subgroup comparison. Please note that no comparison is significantly different (all *p* > 0.05): postop = postoperative at discharge. Quantification of pain according to numeric rating scale (NRS). Quantification of muscle strength according to Janda.

Subgroups showed no significant differences in the time of surgery or length of stay ( *p* = 0.936 and 0.727). Additionally, there were no significant subgroup differences in postoperative neurological status (all *p* > 0.05, Fig. 2 ).

### Within-Group Longitudinal Differences of the Study Group in Neurological Outcome at Postoperative Discharge and Follow-up

As expected, patients experienced significant neurological recovery after PCF. In both subgroups, neck pain decreased significantly postoperatively and during follow-up compared with the preoperative values (C3–C6: *M*_preop_ = 8, *M*_postop_ = 4, chi-square = 1.033, *p* = 0.005; C6–Th1: *M*_preop_ = 7, *M*_postop_ = 3, chi-square = 1.071, *p* = 0.005). The same improvement applied to radicular pain (C3–C6: *M*_preop_ = 8, *M*_postop_ = 1, chi-square = 1.433, *p* < 0.001; C6–Th1: *M*_preop_ = 8, *M*_postop_ = 1, chi-square = 1.500, *p* < 0.001). With regard to paresis, patients with disc herniation in the lower cervical spine showed a significant improvement (C6–Th1: chi-square = 15.929, *p* < 0.001). Specifically, the improvement was detectable at the time of follow-up ( *M*_preop_ = 4, *M*_follow-up_ = 5, chi-square = −0.933, *p* = 0.011), whereas paresis scores in patients with disc herniation in the upper cervical spine did not change significantly throughout the time ( *p* = 0.097). For illustration, see Fig. 1 .

### Within-Group Longitudinal Differences of the Control Group in Neurological Outcome at Postoperative Discharge

Similarly, patients of the control group demonstrated significant neurological recovery following PCF. In both subgroups, preoperative pain decreased significantly postoperatively (C3–C6: *M*_preop_ = 9, *M*_postop_ = 3, *W* = 0.000, *p* < 0.001; C6–Th1: *M*_preop_ = 9, *M*_postop_ = 2, *W* = 1.000, *p <* 0.001). Both subgroups showed a significant postoperative improvement of the preoperative paresis (C3–C6: *M*_preop_ = 4, *M*_follow-up_ = 5, *W* = 28, *p* = 0.008; C6–Th1: *M*_preop_ = 4, *M*_follow-up_ = 5, *W* = 231, *p <* 0.001, see Fig. 2 ).

To provide a quantitative measure of the magnitude of the difference between groups, effect sizes (Hedges's g and Hedges's gav were calculated for all comparisons ( Table 3 ). 17

**Table 3:** Effect sizes and confidence intervals were computed for statistical analyses on group differences

Variable names	Significance ( *p* -value)	Effect size (Hedges's g a )	95% confidence interval	Significance ( *p* -value)	Effect size (Hedges's g)	95% confidence interval
C3–C6 vs. C6–T1	**Study group**			**Control group**		
Age	0.293	0.33	[−0.29, 0.95]	0.255	−0.32	[−0.88, 0.24]
BMI	0.144	−0.46	[−1.08, 0.16]	0.137	**0.59**	[0.01, 1.16]
Neck pain preop.	0.947	0.08	[−0.55, 0.71]	0.089	0.50	[−0.06, 1.06]
Radicular pain preop.	0.864	0.09	[−0.53, 0.72]			
Paresis preop.	**0.033**	0.63	[0, 1.26]	0.172	0.43	[−0.13, 0.99]
Time of surgery	0.987	−0.01	[−0.62, 0.61]	0.936	−0.02	[−0.58, 0.54]
Length of stay	0.869	−0.05	[−0.67, 0.56]	0.727	−0.10	[−0.65, 0.46]
C3–C6						
Neck pain preop. vs. postop.	**0.014**	**1.62**	[0.98, 2.26]	**<0.001**	**3.23**	[2.21, 4.25]
Radicular pain preop. vs. postop.	**<0.001**	**2.97**	[2.17, 3.77]			
Neck pain postop. vs. follow-up	0.249	0.61	[−0.05, 1.26]	n.a.	n.a.	n.a.
Radicular pain postop. vs. follow-up	1.000	−0.15	[−0.79, 0.50]			
Neck pain preop. vs. follow-up	**<0.001**	**2.01**	[1.23, 2.79]	n.a.	n.a.	n.a.
Radicular pain preop. vs. follow-up	**0.001**	**2.54**	[1.70, 3.39]			
Paresis preop. vs. postop.	n.a. ^b^	−0.53	[−1.10, 0.03]	0.080	**−0.93**	[−1.64, −0.22]
Paresis postop. vs. follow-up	n.a. ^b^	−0.25	[−0.89, 0.39]	n.a.	n.a.	n.a.
Paresis preop. vs. follow-up	n.a. ^b^	−0.80	[−1.47, 0.14]	n.a.	n.a.	n.a.
C6–T1						
Neck pain preop. vs. postop.	**0.014**	**2.23**	[1.35, 3.11]	**<0.001**	**3.32**	[2.69, 3.95]
Radicular pain preop. vs. postop.	**<0.001**	**3.61**	[2.40, 4.74]			
Neck pain postop. vs. follow-up	0.771	**0.83**	[0.09, 1.56]	n.a.	n.a.	n.a.
Radicular pain postop. vs. follow-up	1.000	0.00	[−0.7, 0.7]			
Neck pain preop. vs. follow-up	**<0.001**	**2.55**	[1.60, 3.50]	n.a.	n.a.	n.a.
Radicular pain preop. vs. follow-up	**<0.001**	**3.44**	[2.33, 4.55]			
Paresis preop. vs. postop.	0.107	−0.46	[−1.15, 0.22]	**<0.001**	**−0.61**	[−1.02, −0.19]
Paresis postop. vs. follow-up	1.000	−0.45	[−1.15, 0.26]	n.a.	n.a.	n.a.
Paresis preop. vs. follow-up	0.320	**−1.11**	[−1.86, −0.37]	n.a.	n.a.	n.a.

Abbreviations: BMI, body mass index.

a A Hedges' g of e.g., 0.2 is considered a small effect, 0.5 a medium effect, and 0.8 or higher a large effect, indicating that groups differ by 0.2, 0.5, and 0.8 standard deviations, respectively. Accordingly, large effects are in bold. Significances are n.a. = not available in cases where respective data were not acquired or ^b^ no post hoc pairwise comparisons of longitudinal differences between preop. = preoperative, postop. = postoperative and follow-up neurological outcome were performed due to nonsignificant overall test statistics.

### Description of Overall Complications

C3–C6 and C6–T1 subgroups did not differ from each other with regard to blood loss (<100 mL) or revision, neither in the navigated nor in the control group. However, blood loss in the C3–C6 group differed significantly between control and study group (blood loss: χ2(1) = 6.615, *p* = 0.019). Intraoperative causes of hemorrhage are not documented. The reasons for revision surgeries included incomplete decompression of the neuroforaminal stenosis and persistent symptoms that were resistant to conservative treatment.

## Discussion

PCF is a well-established procedure for symptomatic neuroforaminal stenosis due to lateral disc herniation. 2 3 5 7 Compared with ACDF (minimal invasive), PCF preserves cervical mobility and reduces adjacent level disease. 2 18 19 20 While conventional fluoroscopy has traditionally guided intraoperative orientation, 3D navigation is gaining traction due to improved accessibility and superior quality. 11 12 13 21 Fluoroscopy's limitations in visualizing the lower cervical spine are well known. 8 9 Recent studies suggest that 3D navigation facilitates less invasive approaches in PCF by enhancing orientation. 13 22 23 However, no prior studies have examined whether this improved orientation translates into better clinical outcomes, surgery time, or hospital stay duration in the upper versus lower cervical procedures. Thus, we retrospectively investigated these aspects.

3D navigation was successfully used for PCF in our patient cohort, resulting in significant symptom relief. However, we found no significant differences in neurological outcomes, surgery time, length of stay, and revision rates compared with conventional fluoroscopy. Notably, PCF at the lower cervical levels (C6–T1) produced outcomes comparable to upper levels (C3–C6), despite the lower subgroup having a higher average BMI ( *M*_C3–C6_ = 28 and *M*_C6–T1_ = 31, *p* = 0.1848), a factor typically associated with worse outcome. 24 25 Interestingly, surgery time and hospital stay did not differ significantly, suggesting that 3D navigation did not provide a time-saving advantage in this context.

However, a new and speculative hypothesis arises: 3D navigation may be beneficial in selected populations, such as obese patients with short necks, where enhanced anatomical visualization could mitigate challenges associated with conventional fluoroscopy. Although our study did not include a dedicated subgroup analysis, future studies should investigate whether 3D navigation offers specific benefits for patients with challenging anatomical conditions such as obesity or short necks.

In three of 42 cases (7.1%), the herniated disc could not be removed posteriorly, necessitating anterior discectomy and fusion. This aligns with existing data showing that 5 to 7% of PCF patients eventually require revision at the same level. 26 27 In contrast, the control group had a higher revision rate (12.7%) due to persistent postoperative symptoms. Importantly, no wrong-level surgeries occurred. Incorrect level surgery is a serious complication of spinal procedures. 28 29 30 31 A national survey reports up to 50% of spine surgeons perform at least one wrong-level procedure during their careers. 32

We observed significantly higher blood loss (>100 mL) in conventionally operated C3 to C6 patients compared with the 3D-navigated group. However, the absence of detailed volume data limits interpretation. A smaller, less traumatic approach with 3D navigation may explain this difference. Other possible confounders include anticoagulation status, undiagnosed coagulopathies, surgeon experience. These observations are exploratory and should therefore be interpreted with caution. Further prospective studies are needed to confirm these findings.

3D navigation offers precision in cervical spine, particularly in pedicle screw placement. 23 In our experience, 3D navigation improves accuracy but adds complexity. 33 34 It requires advanced imaging, meticulous setup, and surgeon expertise to interpret images and maintain anatomical plausibility. The associated learning curve can initially extend surgical time and increase complication risks. Additionally, technical failures may pose challenges that do not occur with simpler fluoroscopic systems.

## Conclusion

While 3D navigation can help confirm decompression, its technical, logistical, and economic demands should be carefully considered when selecting a surgical method. Future studies should not only evaluate clinical benefits but also systematically assess the cost-effectiveness. Furthermore, 3D navigation should be regarded as an alternative rather than a replacement for fluoroscopy, with its use carefully weighed against technical complexity and radiation exposure, particularly in challenging cervical spine cases.

## Limitations

This study is limited by its retrospective design and the use of a historical control cohort, which may introduce selection and temporal bias. Only navigated cases were performed by a single surgeon. Follow-up in the control group was not standardized, increasing the risk of attention bias. Radiation exposure and intraoperative blood loss could not be quantitatively analyzed due to incomplete documentation. No postoperative imaging was routinely obtained to assess decompression or instability. The learning curve associated with navigation was not evaluated. No cost-effectiveness analysis was performed.
